# Phage Display Technology as a Powerful Platform for Antibody Drug Discovery

**DOI:** 10.3390/v13020178

**Published:** 2021-01-25

**Authors:** Kazuya Nagano, Yasuo Tsutsumi

**Affiliations:** 1 Graduate School of Pharmaceutical Sciences, Osaka University, 1-6 Yamadaoka, Suita, Osaka 565-0871, Japan; 2 Graduate School of Medicine, Osaka University, 2-2 Yamadaoka, Suita, Osaka 565-0871, Japan; 3 The Center for Advanced Medical Engineering and Informatics, Osaka University, 1-6, Yamadaoka, Suita, Osaka 565-0871, Japan

**Keywords:** phage display system, antibody drugs, phage antibody library

## Abstract

Antibody drugs with a high affinity and specificity are effective and safe for intractable diseases, such as cancers and autoimmune diseases. Furthermore, they have played a central role in drug discovery, currently accounting for eight of the top 20 pharmaceutical products worldwide by sales. Forty years ago, clinical trials on antibody drugs that were thought to be a magic bullet failed, partly due to the immunogenicity of monoclonal antibodies produced in mice. The recent breakthrough in antibody drugs is largely because of the contribution of phage display technology. Here, we reviewed the importance of phage display technology as a powerful platform for antibody drug discovery from various perspectives, such as the development of human monoclonal antibodies, affinity enhancement of monoclonal antibodies, and the identification of therapeutic targets for antibody drugs.

## 1. Introduction

In 1985, Smith et al. demonstrated phage display technology using artificial peptide sequences on the N-terminus of a bacteriophage surface protein [1]. Phage display technology allows the construction of libraries in which various peptides and proteins are displayed on the phages, and then the most suitable clone is selected from the library by in vitro panning. Therefore, the clones with an affinity for the target of interest or those with the ability to migrate to the target tissue are enriched from the library. Therefore, the development of phage display technology provides optimal sequences to target peptides or proteins, unlike conventional alanine scanning and other methods, and aids understanding of their molecular evolution [2,3,4,5,6,7]. Although various kinds of molecular display technologies such as ribosome display [8,9], and yeast display technologies [10,11] have been proposed, phage display technology is frequently employed because of a high diversity of molecules that can be displayed and ease of handling [12,13].

**Figure 1:**
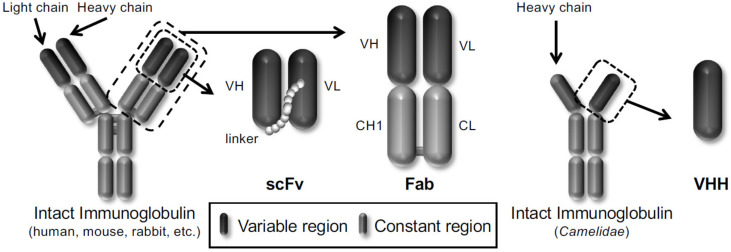
Schematic representation of different types of antibody formats displayed on the phages.

In particular, phage display technology has become a powerful platform for drug discovery in life science because it is easy to produce antibodies in vitro. Winter et al. displayed a single-chain variable fragment (scFv) antibody that consists of the variable heavy chain (VH) and the variable light chain (VL) joined together by a flexible peptide linker in 1990 [14]. Subsequently, several antibody formats, such as scFv, fragment antigen-binding (Fab), and variable fragment (VHH) derived from heavy chain antibodies of *Camelidae*, have been reported to be displayed on the phages (Figure 1). Therefore, the phage display technology allows the construction of various phage antibody libraries such as naïve [15,16], immunized [17,18] and synthetic phage antibody [19,20] and contributes to the current development of antibody drugs. More than 70 phage-derived monoclonal antibodies have entered clinical studies, and 14 of them have been approved for use till May 2020.

Here, we review the importance of phage display technology as a powerful platform for drug discovery, from target discovery to antibody drug development.

Three frequently used antibody formats (scFv, Fab and VHH) are shown.

## 2. Development of Antibody Drugs Using Phage Display Technology

In 1975, Köhler and Milsterin established a method for generating monoclonal antibodies using hybridomas [21]. Since this discovery, monoclonal antibodies with high specificity and affinity to target molecules were expected to be used as magic bullets for various clinical applications [22]. However, clinical trials have largely failed because mouse monoclonal antibodies recognized as heterologous proteins in the human body are highly immunogenic, and they showed reduced efficacy because of human anti-mouse antibodies, so-called HAMAs [23]. This problem has been overcome by antibody engineering. For example, human chimeric antibodies were produced by replacing the Fc sequence of mouse antibodies with that of human antibodies to reduce immunogenicity [24,25]. Moreover, humanized antibodies were produced by replacing their protein sequences with the complementarity-determining region (CDR) of mouse antibodies, which is important for binding to the antigen. Using the same technology, rituximab (Rituxan^®^), infliximab (Remicade^®^), and trastuzumab (Herceptin^®^) were approved in the United States between 1997 and 1998 [24,25]. Nowadays, the production of human antibodies has been established using phage human antibody library [24,25,26] and human antibody-producing transgenic mice [24,25,27].

Taken together, phage display technology has made a significant contribution to antibody engineering. Therefore, we summarized the fundamental mechanisms of antibody engineering and the contributions of phage display technology.

### 2.1. Development of Human Monoclonal Antibodies Using Phage Display Technology

As mentioned above, fewer mouse sequences in the monoclonal antibodies are essential to reduce their immunogenicity in the human body. Therefore, there is an urgent need to create monoclonal antibodies with all-human sequences. In this respect, two approaches to generate such monoclonal antibodies using phage display technology have been established.

The first approach is the use of a phage display-derived human antibody library, in which the antibody repertoire in the human body is displayed in vitro on the phages. For example, Cambridge Antibody Technology (CAT) and Dyax constructed phage display-derived human naïve scFv and Fab antibody libraries by extracting antibody genes from human spleen, peripheral blood lymphocytes, tonsils, and fetal liver [15,16,28,29]. A number of antibodies that are currently approved or in ongoing clinical studies have been isolated from these libraries, such as belimumab against B-lymphocyte stimulator (BLyS) isolated from CAT’s library (Benlysta^®^) [30] and avelumab against programmed death-1 ligand-1 (PD-L1) isolated from Dyax’s library (Bavencio^®^) [31].

The other approach is humanization of mouse monoclonal antibody using the guided selection method [32,33]. Guided selection is based on chain shuffling of variable genes using phage display technology. As an example, in the guided selection procedure using the Fab format, the first step is to clone the variable regions of a mouse antibody to a phagemid vector containing human antibody constant domains, resulting in a chimeric Fab. The next step is to construct a human VL shuffled library by replacing the mouse VL with human VL repertoires and then selecting binders after panning against a target antigen, which results in the selection of human VL paired with the mouse VH. The third step is to construct a human VH shuffled library by replacing the mouse VH with human VH repertoires and then selecting binders after panning against a target antigen, which results in the selection of complete-human Fab clones. Finally, the epitope specificity and affinities of selected human Fabs are confirmed using the appropriate assays. For example, adalimumab against tumor necrosis factor-alpha (TNFα; Humira^®^, clone: D2E7), the first fully human antibody-blockbuster drug, was created from mouse anti-human TNFα (clone: Mab32) as a template using the guided selection method [34].

Therefore, phage display technology contributes to the development of antibody drugs by overcoming immunogenicity as a bottleneck for the magic bullet. The approved antibody drugs derived from phage display technology are shown in Table 1.

### 2.2. In Vitro Affinity Maturation of Monoclonal Antibodies Using Phage Display Technology

Antibodies are effective and safe drugs that can achieve complete remission for intractable diseases, such as cancers and autoimmune diseases [57,58] because they have a high affinity and specificity for their antigens. However, many antibodies, isolated from naïve phage, antibody libraries with an antibody repertoire for any antigen have low affinity for the antigens because somatic hypermutations did not occur. Therefore, in order to use antibodies with low affinity for the antigens as research tools for therapeutics and diagnostics, it is necessary to enhance the affinity for the antigens. Phage display technology is commonly used to improve the affinity for antigens, which is important for high antibody efficacy. A number of antibody drugs such as moxetumomab pasudotox against CD22 (Lumoxiti^®^) [52] and ranibizumab against vascular endothelial growth factor A (VEGFA) (Lucentis^®^) [35] have been manufactured by in vitro affinity maturation using phage display technology. In this approach, gene libraries are constructed with mutations in the antigen-binding regions such as VL and VH, the libraries are displayed on the phages, and the antibodies with high affinity for the antigens are then isolated by biopanning. In order to construct gene libraries with mutations, there are two major approaches: (1) Random mutagenesis and (2) site-specific mutagenesis.

In random mutagenesis, mutagenesis is induced by anticancer agents [59], radiation [59], or by gene recombination with chain shuffling [14,60,61]. Among these techniques, error-prone polymerase chain reaction (error-prone PCR) is often employed [62]. This approach leverages the natural error rate of a low-fidelity DNA polymerase. In this approach, the affinity of monoclonal antibodies is enhanced using the following steps. Mutations in the CDRs of the ideal monoclonal antibody clone for affinity maturation are randomly introduced by error-prone PCR. From these mutation libraries, the clones that strongly bind to the antigen are enriched by biopanning. By repeating these steps, antibody clones with a higher affinity than the template antibody can be generated. The method of constructing mutation libraries by error-prone PCR and selecting the antibody clone with high affinity by biopanning has been applied to various antibodies, and their affinities have been improved. In particular, using the error-prone DNA shuffling method combined with DNA shuffling, the affinity of anti-fluorescein scFv was improved with dissociation constant (KD) of up to 50 fM and slower dissociation kinetics (half-time > 5 days) than those for the streptavidin-biotin complex [63]. As random mutagenesis using methods such as error-prone PCR is not site-specific and randomly causes mutations throughout the sequence, the introduction of logically inconceivable mutations can enhance the interaction and increase the stability of the structure of the antibody. Conversely, there are concerns that the three-dimensional structure of the antibody contact region may be disrupted by introducing random mutations.

Against this background, site-specific mutagenesis can be used to theoretically estimate the sites to be mutated from the structural data of the antibody [64] or the hotspot sequence of the somatic hypermutation [65,66], and then mutate them into a sequence encoding 20 different amino acids using PCR with mutagenic primers [67,68]. Thus, the antibody with improved affinity can be efficiently prepared without disrupting the structure of the antibody, if the sites in which mutations are introduced are appropriate. For example, Chowdhury et al. constructed a library of randomly mutated hotspot sequences of somatic hypermutations, such as A/G-G-C/T-A/T and AGT in the VL CDR3 of anti-mesothelin scFv, and enriched high-affinity antibodies for mesothelin by biopanning. A series of procedures resulted in the successful isolation of the higher-affinity scFvs with KD of several hundred pM [65].

Therefore, using the phage display technology, high-affinity antibodies can be efficiently isolated from various types of mutagenesis libraries, which have their own advantages and disadvantages and complement each other (Table 2).

**Figure 2:**
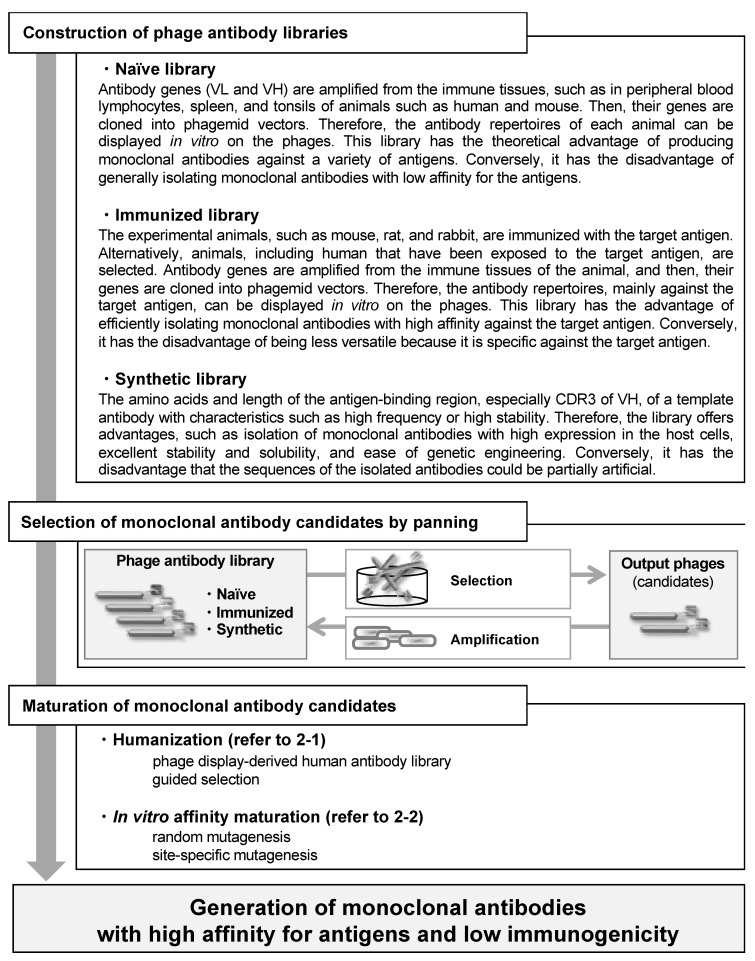
Flow chart outlining the sequence of events from construction of phage antibody libraries to generation of monoclonal antibodies with high affinity for antigens and low immunogenicity.

Taken together, monoclonal antibodies with high affinity for antigens and low immunogenicity have been produced using phage display technology, according to the flow chart in Figure 2. Furthermore, as this technology can be applied to a variety of proteins as well as antibody drugs, it is a versatile and powerful platform for drug discovery.

The characteristics of three frequently used phage antibody libraries are mentioned here. For other processes, refer to the respective sections in the text.

## 3. Target Discovery Using Phage Display Technology

By developing and using the technologies described in the previous section, antibody drugs currently account for eight of the top 20 pharmaceutical products worldwide by sales (Table 3). Furthermore, many monoclonal antibodies are in ongoing clinical trials [69,70]. On the other hand, as the number of targets for these antibody drug candidates is small, many monoclonal antibody clones have been developed for the same promising targets. For example, among the antibodies approved or clinically tested in Japan, four clones including nivolumab (Opdivo^®^) [71,72], pembrolizumab (Keytruda^®^) [73,74], spartalizumab [75,76] and cemiplimab [77,78] have been developed for programmed death 1 (PD-1), and three clones including avelumab (Bavencio^®^) [79,80], atezolizumab (Tecentriq^®^) [81,82] and durvalumab (Imfinzi^®^) [83,84] have been developed for PD-L1. It has recently been noted that the targets of antibody drugs are dwindling. Therefore, it is essential to identify novel targets for the future development of antibody drugs. In this section, we discuss examples of utilizing phage display technology for the discovery of novel therapeutic targets.

### 3.1. Search for Antigens that React with Autoantibodies Using Phage Display Technology

Autoimmune diseases are intractable diseases in which the immune system fails to function properly and attacks its own body tissues. The causes of autoimmune disease, such as rheumatoid arthritis (RA) [85,86], systemic lupus erythematosus (SLE) [87,88] and multiple sclerosis (MS) [89,90] remain unknown. Although, the recent development of biologics, mainly antibody drugs, has partially improved the outcomes of diseases, such as RA [91,92,93,94], the prognosis for these diseases remains poor. Therefore, understanding the molecular pathogenesis of autoimmune diseases and searching for new therapeutic targets is essential for the development of new therapies.

Antibodies to self-antigens produced in autoimmune diseases can be used as biomarkers. For example, in vasculitis, the number of antibodies to myeloperoxidase and proteinase 3 in neutrophils was clinically tested [95,96]. In SLE, the number of antibodies to double-stranded DNA and small nuclear ribonucleoprotein in the nucleus has also been clinically tested [97,98]. Therefore, the search for antigens that bind to autoantibodies in autoimmune diseases is a promising approach to elucidate molecular pathogenesis and target identification.

In this respect, self-antigens specific for autoimmune diseases can be efficiently explored using phage display technology. To achieve this, a complementary DNA (cDNA) phage display library is first constructed based on mRNA extracted from biological tissues. Next, the phage clones that bind to the antibodies collected from blood and other sources from patients with autoimmune diseases are enriched by biopanning using this library. Finally, by analyzing the sequences of cDNA in the eluted phages, self-antigens are identified using high-throughput technologies. For example, in the case of MS, self-antigens binding to IgG in cerebrospinal fluid and serum of patients have been explored by constructing cDNA libraries from the human brain. As a result, 14 different self-antigens have been identified, including DEAD-box helicase 24 [99]. Moreover, similar approaches have also been applied to autoimmune diseases such as RA [100] and SLE [101].

Besides autoimmune diseases, cancer antigens have also been identified from autoantibodies in cancer patients. For example, in head and neck cancers, the cancer-specific antigens displayed on the phages were enriched by subtraction biopanning. Validation of 21 clones demonstrated L23 to be a novel cancer antigen, which is highly expressed in head and neck cancers compared that in normal keratinocytes. Knockdown of L23 inhibited proliferation, invasion, and cell survival, whereas its overexpression showed opposite effects [102]. Furthermore, similar approaches have also been applied to various types of cancers, such as breast [103] and prostate cancers [104], and paraneoplastic neurological syndrome [105].

### 3.2. High-Throughput Validation of Therapeutic Target Candidates Using Phage Display Technology

In current drug discovery methods, it is important to identify not only therapeutic targets but also biomarkers for understanding pathological conditions, including approaches such as companion diagnostics. An approach to identify these molecules involves comprehensive omics studies, such as genomics [106,107] and transcriptomics [107,108] have been attracting attention. In particular, proteomics is a large-scale study in which proteins, end products of the central dogma, are comprehensively analyzed, and proteomics plays a central role in post-genome research [109,110]. An approach to identify therapeutic targets and biomarkers involves comparing proteins expressed in cells or tissues of healthy and diseased cases, called disease proteomics, is commonly used [111,112]. This research area has made significant progress because even small numbers of differentially expressed proteins can be efficiently identified by improving the performance of mass spectrometry [113,114]. Therefore, the remaining issue for this research area is the high-throughput validation of these disease-related proteins.

In this respect, monoclonal antibodies with high a affinity and specificity for the antigen proteins have been commonly used [115,116,117]. Techniques using monoclonal antibodies, such as enzyme-linked immunosorbent assay, western blotting, fluorescent imaging, and tissue microarray (TMA) staining are extremely useful for examining the function and distribution of proteins [118,119]. Usually, monoclonal antibodies are generated using hybridomas; however, this approach is laborious and time-consuming, and it requires a large number of recombinant antigens. Furthermore, protein production using this approach often requires gene engineering for heterologous expression, which takes time for optimization. Therefore, it is impractical to produce monoclonal antibodies against the multiple candidate proteins identified when using a proteomics approach for protein selection. To address this issue, an approach called antibody proteomics technology has been developed, which uses a phage antibody library and TMA analysis to rapidly and comprehensively isolate monoclonal antibodies against candidate proteins for the identification of potential biomarkers and therapeutic targets [120]. In this section, we describe the development and evaluation of this novel technology.

First, in order to produce monoclonal antibodies against a large number of disease-related proteins efficiently, we focused on a naïve phage antibody library that can produce monoclonal antibodies against various types of antigens in vitro. Thus, we previously constructed an improved naïve phage antibody library by designing a PCR primer set that allowed for comprehensive amplification of the VH and VL genes, so that rapid isolation of monoclonal antibodies against multiple target proteins can be performed in vitro [121,122]. Subsequently, we attempted to construct a monoclonal antibody preparation method using the proteins (in the order of ng) that can be recovered directly from two-dimensional differential in-gel electrophoresis (2D-DIGE) gels, which are commonly used in proteomics studies, without the preparation of recombinant protein as an antigen. Monoclonal antibodies can be isolated even from small amounts of antigen (only 0.5 ng in the case of kinase insert domain receptor protein) using a nitrocellulose membrane with excellent protein adsorption capacity as an immobilized carrier for antigen proteins, which is called nitrocellulose membrane panning method [120]. These processes allowed us to establish a method for rapid antibody preparation in vitro for many candidates identified using 2D-DIGE analysis. Finally, in order to efficiently validate the candidates in a large number of clinical samples, we focused on TMA using clinical tissue sections from multiple cases mounted on a single glass slide. Immunohistochemical staining of TMA with monoclonal antibodies against the antigen of interest allowed us to elucidate the expression profiles of a large number of cases in a single analysis. In addition, we could also analyze the correlation of expression profiles with clinical information, including age, sex, medical history, and medications. By optimizing the conditions, we established a method for immunohistochemical staining of TMA using monoclonal antibodies displayed on the phage, without the preparation of recombinant monoclonal antibodies [120]. By combining these techniques, we could perform the entire process from proteome analysis to identification of therapeutic targets and biomarker proteins in approximately one month.

**Figure 3:**
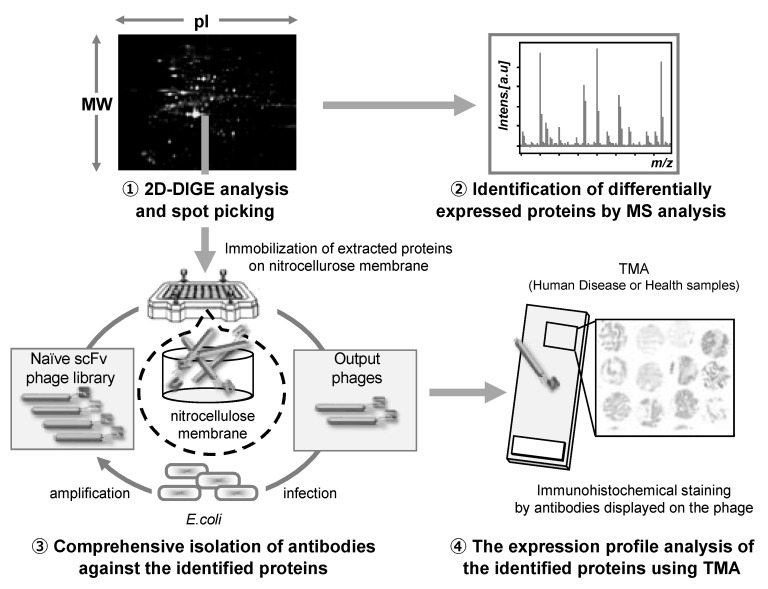
Schematic illustration of the antibody proteomics system. (1) Candidate proteins are detected using two-dimensional differential in-gel electrophoresis (2D-DIGE) and (2) identified by mass spectrometry analysis (MSA). Simultaneously, (3) monoclonal antibodies against all the proteins identified by 2D-DIGE are produced using a phage antibody library. Finally, (4) the proteins are validated as biomarkers and therapeutic targets using tissue microarray (TMA). Therefore, using this technology, the candidate proteins can be comprehensively validated, and the most useful proteins can be selected.

The antibody proteomics technology comprises four stages: (1) Search for disease-related proteins by proteomics-based analysis using 2D-DIGE, (2) identification of the candidate proteins using mass spectrometry analysis, (3) isolation of monoclonal antibodies against the candidate proteins using a phage antibody library, and (4) validation of the candidate proteins using TMA analysis (Figure 3).

Hence, this technology accelerates the identification of proteins that are potentially useful as biomarkers or therapeutic targets. Moreover, it could be an alternative to the conventional approach in which prioritized proteins are validated one by one among the identified proteins, and it can become a fundamental system for drug discovery.

To evaluate the practicality of this technology, we applied it to the search for therapeutic targets for breast cancer [120,123]. We identified a novel therapeutic target that is specifically expressed in the testes of normal tissues and refractory cases of breast cancer (triple-negative breast cancer) called Eph receptor A10 (EphA10) [124,125,126]. EphA10 expression was also shown to be correlated with breast cancer stage and lymph node metastasis, suggesting that it is a promising target for breast cancer therapy [127]. In order to demonstrate the proof of concept that inhibition of EphA10 specifically expressed in breast cancers induces an anti-tumor effect, we generated a neutralizing monoclonal antibody against EphA10 and then analyzed the therapeutic effect in a xenograft mouse model. Analysis of tumor volumes showed that anti-EphA10 neutralizing monoclonal antibody inhibited tumor growth in a concentration-dependent manner [123,128]. In addition, EphA10 was highly expressed not only in breast cancer but also in prostate cancer [129], suggesting that EphA10 could be a promising target for various kinds of tumors.

We also applied this technology to the search of biomarkers of metastasis in lung cancers, an important prognostic factor in cancers, and found that the expression levels of oxysterol-binding proteins, such as oxysterol-binding protein-related protein 5 (OSBPL5) and calumenin (CALU), were significantly higher in positive cases of lymph node metastasis than in negative cases [130]. Interestingly, 80 % of co-expression cases with both proteins were positive for lymph node metastasis, whereas approximately 50–60 % of cases with expression of at least one of the two proteins were positive for lymph node metastasis. Furthermore, invasion assays showed that the lung cancer cell lines in which these genes were overexpressed or knocked down significantly enhanced or inhibited the invasion capacity, suggesting that both proteins had the ability to promote lung cancer cell invasion. Therefore, OSBPL5 and CALU play a role in lymph node metastasis by enhancing the invasiveness of lung cancer cells.

Finally, we applied the technology to the search for biomarkers for companion diagnostics. While, cisplatin has been commonly used as a major anticancer agent against malignant mesothelioma, it has many adverse side effects. In a previous study, we identified that the malignant mesothelioma cell lines in which the Annexin A4 gene was overexpressed or knocked down showed significantly inhibition or enhancement of the cytotoxicity of cisplatin, demonstrating that Annexin A4 is involved in cisplatin resistance [131].

In this manner, antibody proteomics technology has enabled selection of promising proteins from disease-related proteins based on scientific evidence. Consequently, it is expected that antibody proteomics technology will aid identification of a large number of therapeutic targets and biomarker proteins for the development of novel diagnostic and therapeutic agents.

## 4. Conclusions and Prospects

In this review, we discussed the significant contribution of phage display technology for the development of antibody drugs, which play a central role in drug discovery from various perspectives.

In addition to the ones mentioned above, bispecific antibodies that recognize two antigens in a single antibody format are currently being commercialized [132,133,134]. Phage display technology is also used as an essential technology for the development of bispecific antibodies [135,136,137]. Furthermore, this technology is used for the development of antibody-mimicking peptides, microantibodies, as potential next-generation biologics [138,139]. Therefore, it is expected that phage display technology will continue to be a powerful platform to lead innovative drug discovery in the future [140].

## Figures and Tables

**Table 1:** A list of approved antibody drugs derived from phage display technology.

Product Name	Nonproprietary Name	Target Antigen	First Application	Approved Year	Special Note on Phage Display Technology
Humira^®^	Adalimumab	TNFα	RA	2002	Humanization using guided selection method [34]
Lucentis^®^	Ranibizumab	VEGFA	nAMD	2006	In vitro affinity maturation [35].
Benlysta^®^	Belimumab	BLyS	SLE	2011	Isolation from CAT’s library (human naïve scFv library) [30]
ABthrax^®^	Raxibacumab	*Bacillus anthracis* PA	Inhaled anthrax	2012	Isolation from CAT’s library (human naïve scFv library) [36]
Cyramza^®^	Ramucirumab	VEGFR2	GC NSCLC	2014	Isolation from Dyax’s library (human naïve Fab library) [37,38]
Portrazza^®^	Necitumumab	EGFR	NSCLC	2015	Isolation from Dyax’s library (human naïve Fab library) [16,39]
Taltz^®^	Ixekizumab	IL-17A	Psoriasis	2016	Isolation from mouse immune Fab library [40,41]
Tecentriq^®^	Atezolizumab	PD-L1	UC NSCLC	2016	Isolation from Genentech’s library (human naïve library) [42,43]
Bavencio^®^	Avelumab	PD-L1	MCC	2017	Isolation from Dyax’s library (human naïve Fab library) [31,44]
Tremfya^®^	Guselkumab	IL-23	Psoriasis	2017	Isolation from HuCAL GOLD^®^ library (Synthetic Fab library) [45,46]
Cablivi^®^	Caplacizumab	vWF	aTTP	2018	Isolation from Camelidae-derived nanobody library [47,48,49]
Gamifant^®^	Emapalumab	IFNγ	HLH	2018	Isolation from CAT’s library (human naïve scFv library) [50,51]
Lumoxiti^®^	Moxetumomab pasudotox	CD22	HCL	2018	In vitro affinity maturation [52,53,54].
Takhzyro^®^	Lanadelumab	pKal	HAE	2018	Isolation from Dyax’s library (human naïve Fab library) [55,56].

Abbreviations: TNFα: tumor necrosis factor-alpha, VEGFA: vascular endothelial growth factor A, BLyS: B-lymphocyte stimulator, *Bacillus anthracis* PA: *Bacillus anthracis* protective antigen, VEGFR2: vascular endothelial growth factor receptor 2, EGFR: epidermal growth factor receptor, IL-17A: interleukin-17A, PD-L1: programmed death-1 ligand-1, IL-23: interleukin-23, vWF: von Willebrand factor, IFNγ: interferon-gamma, pKal: plasma kallikrein, RA: rheumatoid arthritis, nAMD: neovascular age-related macular degeneration, SLE: systemic lupus erythematosus, GC: gastric carcinoma, NSCLC: non-small cell lung carcinoma, UC: urothelial carcinoma, MCC: Merkel cell carcinoma, aTTP: acquired thrombotic thrombocytopenic purpura, HLH: hemophagocytic lymphohistiocytosis, HCL: hairy cell leukemia, HAE: hereditary angioedema, Fab: fragment antigen-binding, scFv: single-chain variable fragment, CAT: Cambridge Antibody Technology.

**Table 2:** Advantages and disadvantages of random mutagenesis and site-specific mutagenesis for constructing gene libraries with mutations.

	Advantages	Disadvantages
**Random mutagenesis**	The introduction of logically inconceivable mutation can enhance the interaction of antigen-antibody and increase the stability of the structure of the antibody.	The three-dimensional structure of the antibody contact region may be disrupted by introducing random mutations.
**Site-specific mutagenesis**	The antibody with higher affinity could be efficiently prepared without disrupting the structure of the antibody.	The introduction of logically inconceivable mutations cannot be established.

**Table 3:** The top 20 pharmaceutical products worldwide by sales in 2019.

	Product Name	Nonproprietary Name	Drug Format	Target Antigen	Main Application	Sales Amount ^1^
1	Humira^®^	Adalimumab	Antibody	TNFα	RA ^2^	26.85
2	Eliquis^®^	Apixaban	Organic compound	Fxa	Thrombocytopenia	13.47
3	Keytruda^®^	Pembrolizumab	Antibody	PD-1	Cancer	11.36
4	Xarelto^®^	Rivaroxaban	Organic compound	Fxa	Thrombocytopenia	10.38
5	Lantus^®^	Insulin glargine	Peptide	Insulin receptor	Diabetes	10.01
6	Enbrel^®^	Etanercept	Fc fusion protein ^3^	TNFα	RA	9.71
7	Stelara^®^	Ustekinumab	Antibody	IL-12/23	Psoriasis	8.79
8	Opdivo^®^	Nivolumab	Antibody	PD-1	Cancer	8.03
9	Januvia^®^	Sitagliptin	Organic compound	DPP-4	Diabetes	7.47
10	NovoRapid^®^	Insulin aspart	Peptide	Insulin receptor	Diabetes	7.39
11	Trulicity^®^	Dulaglutide	Fc fusion protein ^4^	GLP-1 receptor	Diabetes	7.30
12	Remicade^®^	Infliximab	Antibody	TNFα	RA	6.96
13	Avastin^®^	Bevacizumab	Antibody	VEGF	Cancer	6.47
14	Rituxan^®^	Rituximab	Antibody	CD20	Cancer	5.90
15	Humalog^®^	Insulin lispro	Peptide	Insulin receptor	Diabetes	5.83
16	Herceptin^®^	Trastuzumab	Antibody	HER2	Cancer	5.72
17	Imbruvica^®^	Ibrutinib	Organic compound	Tyrosine kinase	Cancer	5.69
18	Symbicort^®^	Budesonide	Organic Compound	-	Asthma	5.60
19	Revlimid^®^	Lenalidomide	Organic Compound	-	Cancer	5.59
20	Ibrance^®^	Palbociclib	Organic Compound	CDK4/6	Cancer	5.54

^1^ Multiplied by $1 billion, ^2^ rheumatoid arthritis, ^3^ TNF receptor II and IgG1 Fc Fusion protein, ^4^ GLP-1 analog and IgG4 Fc fusion protein (modification of the investigation by IQVIA, USA). **Abbreviations:** TNFα: tumor necrosis factor-alpha; Fxa: factor Xa; PD-1: programmed death 1; IL-12: interleukin-12; DPP-4: dipeptidyl peptidase-4; VEGF: vascular endothelial growth factor; GLP-1: glucagon-like peptide-1; CDK: cyclin-dependent protein kinase; HER2: human epidermal growth factor receptor 2.

## References

[1] Smith G.P. (1985). Filamentous fusion phage: Novel expression vectors that display cloned antigens on the virion surface. Science.

[2] Widersten M., Mannervik B. (1995). Glutathione transferases with novel active sites isolated by phage display from a library of random mutants. J. Mol. Biol..

[3] Jiang B., Liu W., Qu H., Meng L., Song S., Ouyang T., Shou C. (2005). A novel peptide isolated from a phage display peptide library with trastuzumab can mimic antigen epitope of HER-2. J. Biol. Chem..

[4] Mukai Y., Sugita T., Yamato T., Yamanada N., Shibata H., Imai S., Abe Y., Nagano K., Nomura T., Tsutsumi Y. (2006). Creation of novel Protein Transduction Domain (PTD) mutants by a phage display-based high-throughput screening system. Biol. Pharm. Bull..

[5] Shibata H., Yoshioka Y., Ohkawa A., Minowa K., Mukai Y., Abe Y., Taniai M., Nomura T., Kayamuro H., Nabeshi H. (2008). Creation and X-ray structure analysis of the tumor necrosis factor receptor-1-selective mutant of a tumor necrosis factor-alpha antagonist. J. Biol. Chem..

[6] Yu H., Segers F., Sliedregt-Bol K., Bot I., Woltman A.M., Boross P., Verbeek S., Overkleeft H., van der Marel G.A., van Kooten C. (2013). Identification of a novel CD40 ligand for targeted imaging of inflammatory plaques by phage display. FASEB J..

[7] Altmann A., Sauter M., Roesch S., Mier W., Warta R., Debus J., Dyckhoff G., Herold-Mende C., Haberkorn U. (2017). Identification of a Novel ITGalphavbeta6-Binding Peptide Using Protein Separation and Phage Display. Clin. Cancer Res..

[8] Yan X., Xu Z. (2006). Ribosome-display technology: Applications for directed evolution of functional proteins. Drug Discov. Today.

[9] Kunamneni A., Ogaugwu C., Bradfute S., Durvasula R. (2020). Ribosome Display Technology: Applications in Disease Diagnosis and Control. Antibodies.

[10] Feldhaus M.J., Siegel R.W. (2004). Yeast display of antibody fragments: A discovery and characterization platform. J. Immunol. Methods.

[11] Traxlmayr M.W., Obinger C. (2012). Directed evolution of proteins for increased stability and expression using yeast display. Arch. Biochem. Biophys..

[12] Kristensen P., Ravn P., Jensen K.B., Jensen K. (2000). Applying phage display technology in aging research. Biogerontology.

[13] Wang Y., Gao S., Lv J., Lin Y., Zhou L., Han L. (2019). Phage Display Technology and its Applications in Cancer Immunotherapy. Anticancer Agents Med. Chem..

[14] Marks J.D., Griffiths A.D., Malmqvist M., Clackson T.P., Bye J.M., Winter G. (1992). By-passing immunization: Building high affinity human antibodies by chain shuffling. Biotechnology.

[15] Cordeiro M.F. (2003). Technology evaluation: Lerdelimumab, Cambridge Antibody Technology. Curr. Opin. Mol. Ther..

[16] Garnock-Jones K.P. (2016). Necitumumab: First Global Approval. Drugs.

[17] Chowdhury P.S., Viner J.L., Beers R., Pastan I. (1998). Isolation of a high-affinity stable single-chain Fv specific for mesothelin from DNA-immunized mice by phage display and construction of a recombinant immunotoxin with anti-tumor activity. Proc. Natl. Acad. Sci. USA.

[18] Silence K., Dreier T., Moshir M., Ulrichts P., Gabriels S.M., Saunders M., Wajant H., Brouckaert P., Huyghe L., Van Hauwermeiren T. (2014). ARGX-110, a highly potent antibody targeting CD70, eliminates tumors via both enhanced ADCC and immune checkpoint blockade. MAbs.

[19] Rothe C., Urlinger S., Lohning C., Prassler J., Stark Y., Jager U., Hubner B., Bardroff M., Pradel I., Boss M. (2008). The human combinatorial antibody library HuCAL GOLD combines diversification of all six CDRs according to the natural immune system with a novel display method for efficient selection of high-affinity antibodies. J. Mol. Biol..

[20] Prassler J., Thiel S., Pracht C., Polzer A., Peters S., Bauer M., Norenberg S., Stark Y., Kolln J., Popp A. (2011). HuCAL PLATINUM, a synthetic Fab library optimized for sequence diversity and superior performance in mammalian expression systems. J. Mol. Biol..

[21] Kohler G., Milstein C. (1975). Continuous cultures of fused cells secreting antibody of predefined specificity. Nature.

[22] Raso V. (1990). Antibodies in diagnosis and therapy. The magic bullet--nearing the century mark. Semin. Cancer Biol..

[23] Peterson N.C. (1996). Recombinant antibodies: Alternative strategies for developing and manipulating murine-derived monoclonal antibodies. Lab. Anim. Sci..

[24] Sandhu J.S. (1992). Protein engineering of antibodies. Crit. Rev. Biotechnol..

[25] Maynard J., Georgiou G. (2000). Antibody engineering. Annu. Rev. Biomed. Eng..

[26] Frenzel A., Schirrmann T., Hust M. (2016). Phage display-derived human antibodies in clinical development and therapy. MAbs.

[27] Green L.L. (2014). Transgenic mouse strains as platforms for the successful discovery and development of human therapeutic monoclonal antibodies. Curr. Drug Discov. Technol..

[28] Vaughan T.J., Williams A.J., Pritchard K., Osbourn J.K., Pope A.R., Earnshaw J.C., McCafferty J., Hodits R.A., Wilton J., Johnson K.S. (1996). Human antibodies with sub-nanomolar affinities isolated from a large non-immunized phage display library. Nat. Biotechnol..

[29] De Haard H.J., van Neer N., Reurs A., Hufton S.E., Roovers R.C., Henderikx P., de Bruine A.P., Arends J.W., Hoogenboom H.R. (1999). A large non-immunized human Fab fragment phage library that permits rapid isolation and kinetic analysis of high affinity antibodies. J. Biol. Chem..

[30] Baker K.P., Edwards B.M., Main S.H., Choi G.H., Wager R.E., Halpern W.G., Lappin P.B., Riccobene T., Abramian D., Sekut L. (2003). Generation and characterization of LymphoStat-B, a human monoclonal antibody that antagonizes the bioactivities of B lymphocyte stimulator. Arthritis Rheum.

[31] Kim E.S. (2017). Tivozanib: First Global Approval. Drugs.

[32] Almagro J.C., Fransson J. (2008). Humanization of antibodies. Front. BioSci..

[33] Kim S.J., Hong H.J. (2012). Humanization by guided selections. Methods Mol. Biol..

[34] Wang Z., Wang Y., Li Z., Li J., Dong Z. (2000). Humanization of a mouse monoclonal antibody neutralizing TNF-alpha by guided selection. J. Immunol. Methods.

[35] Chen Y., Wiesmann C., Fuh G., Li B., Christinger H.W., McKay P., de Vos A.M., Lowman H.B. (1999). Selection and analysis of an optimized anti-VEGF antibody: Crystal structure of an affinity-matured Fab in complex with antigen. J. Mol. Biol..

[36] Mazumdar S. (2009). Raxibacumab. MAbs.

[37] Lu D., Jimenez X., Zhang H., Bohlen P., Witte L., Zhu Z. (2002). Selection of high affinity human neutralizing antibodies to VEGFR2 from a large antibody phage display library for antiangiogenesis therapy. Int. J. Cancer.

[38] Lu D., Shen J., Vil M.D., Zhang H., Jimenez X., Bohlen P., Witte L., Zhu Z. (2003). Tailoring in vitro selection for a picomolar affinity human antibody directed against vascular endothelial growth factor receptor 2 for enhanced neutralizing activity. J. Biol. Chem..

[39] Li S., Kussie P., Ferguson K.M. (2008). Structural basis for EGF receptor inhibition by the therapeutic antibody IMC-11F8. Structure.

[40] Liu L., Lu J., Allan B.W., Tang Y., Tetreault J., Chow C.K., Barmettler B., Nelson J., Bina H., Huang L. (2016). Generation and characterization of ixekizumab, a humanized monoclonal antibody that neutralizes interleukin-17A. J. Inflamm. Res..

[41] Markham A. (2016). Ixekizumab: First Global Approval. Drugs.

[42] Herbst R.S., Soria J.C., Kowanetz M., Fine G.D., Hamid O., Gordon M.S., Sosman J.A., McDermott D.F., Powderly J.D., Gettinger S.N. (2014). Predictive correlates of response to the anti-PD-L1 antibody MPDL3280A in cancer patients. Nature.

[43] Markham A. (2016). Atezolizumab: First Global Approval. Drugs.

[44] Kim E.S. (2017). Avelumab: First Global Approval. Drugs.

[45] Markham A. (2017). Guselkumab: First Global Approval. Drugs.

[46] Boehncke W.H., Brembilla N.C., Nissen M.J. (2020). Guselkumab: The first selective IL-23 inhibitor for active psoriatic arthritis in adults. Expert Rev. Clin. Immunol..

[47] Ulrichts H., Silence K., Schoolmeester A., de Jaegere P., Rossenu S., Roodt J., Priem S., Lauwereys M., Casteels P., Van Bockstaele F. (2011). Antithrombotic drug candidate ALX-0081 shows superior preclinical efficacy and safety compared with currently marketed antiplatelet drugs. Blood.

[48] Peyvandi F., Scully M., Kremer Hovinga J.A., Cataland S., Knobl P., Wu H., Artoni A., Westwood J.P., Mansouri Taleghani M., Jilma B. (2016). Caplacizumab for Acquired Thrombotic Thrombocytopenic Purpura. N. Engl. J. Med..

[49] Duggan S. (2018). Caplacizumab: First Global Approval. Drugs.

[50] Al-Salama Z.T. (2019). Emapalumab: First Global Approval. Drugs.

[51] Locatelli F., Jordan M.B., Allen C., Cesaro S., Rizzari C., Rao A., Degar B., Garrington T.P., Sevilla J., Putti M.C. (2020). Emapalumab in Children with Primary Hemophagocytic Lymphohistiocytosis. N. Engl. J. Med..

[52] Ho M., Nagata S., Pastan I. (2006). Isolation of anti-CD22 Fv with high affinity by Fv display on human cells. Proc. Natl. Acad. Sci. USA.

[53] Kreitman R.J., Pastan I. (2011). Antibody fusion proteins: Anti-CD22 recombinant immunotoxin moxetumomab pasudotox. Clin. Cancer Res..

[54] Dhillon S. (2018). Moxetumomab Pasudotox: First Global Approval. Drugs.

[55] Kenniston J.A., Faucette R.R., Martik D., Comeau S.R., Lindberg A.P., Kopacz K.J., Conley G.P., Chen J., Viswanathan M., Kastrapeli N. (2014). Inhibition of plasma kallikrein by a highly specific active site blocking antibody. J. Biol. Chem..

[56] Syed Y.Y. (2018). Lanadelumab: First Global Approval. Drugs.

[57] Melsheimer R., Geldhof A., Apaolaza I., Schaible T. (2019). Remicade((R)) (infliximab): 20 years of contributions to science and medicine. Biologics.

[58] Emancipator K. (2020). Keytruda and PD-L1: A Real-World Example of Co-development of a Drug with a Predictive Biomarker. AAPS J..

[59] DeMarini D.M. (2020). The mutagenesis moonshot: The propitious beginnings of the environmental mutagenesis and genomics society. Environ. Mol. Mutagen..

[60] Stemmer W.P. (1994). Rapid evolution of a protein in vitro by DNA shuffling. Nature.

[61] Lantto J., Jirholt P., Barrios Y., Ohlin M. (2002). Chain shuffling to modify properties of recombinant immunoglobulins. Methods Mol. Biol..

[62] Labrou N.E. (2010). Random mutagenesis methods for in vitro directed enzyme evolution. Curr. Protein Pept. Sci..

[63] Boder E.T., Midelfort K.S., Wittrup K.D. (2000). Directed evolution of antibody fragments with monovalent femtomolar antigen-binding affinity. Proc. Natl. Acad. Sci. USA.

[64] Yamashita T., Mizohata E., Nagatoishi S., Watanabe T., Nakakido M., Iwanari H., Mochizuki Y., Nakayama T., Kado Y., Yokota Y. (2019). Affinity Improvement of a Cancer-Targeted Antibody through Alanine-Induced Adjustment of Antigen-Antibody Interface. Structure.

[65] Chowdhury P.S., Pastan I. (1999). Improving antibody affinity by mimicking somatic hypermutation in vitro. Nat. Biotechnol..

[66] McConnell A.D., Do M., Neben T.Y., Spasojevic V., MacLaren J., Chen A.P., Altobell L., Macomber J.L., Berkebile A.D., Horlick R.A. (2012). High affinity humanized antibodies without making hybridomas; immunization paired with mammalian cell display and in vitro somatic hypermutation. PLoS ONE.

[67] Kawamura M., Shibata H., Kamada H., Okamoto T., Mukai Y., Sugita T., Abe Y., Imai S., Nomura T., Nagano K. (2006). A novel method for construction of gene fragment library to searching epitopes. Biochem. Biophys. Res. Commun..

[68] Kamada H., Okamoto T., Kawamura M., Shibata H., Abe Y., Ohkawa A., Nomura T., Sato M., Mukai Y., Sugita T. (2007). Creation of novel cell-penetrating peptides for intracellular drug delivery using systematic phage display technology originated from Tat transduction domain. Biol. Pharm. Bull..

[69] Glennie M.J., Johnson P.W. (2000). Clinical trials of antibody therapy. Immunol. Today.

[70] Zhao P., Zhang Y., Li W., Jeanty C., Xiang G., Dong Y. (2020). Recent advances of antibody drug conjugates for clinical applications. Acta Pharm. Sin. B.

[71] Wolchok J.D., Kluger H., Callahan M.K., Postow M.A., Rizvi N.A., Lesokhin A.M., Segal N.H., Ariyan C.E., Gordon R.A., Reed K. (2013). Nivolumab plus ipilimumab in advanced melanoma. N. Engl. J. Med..

[72] Deeks E.D. (2014). Nivolumab: A review of its use in patients with malignant melanoma. Drugs.

[73] Bagcchi S. (2014). Pembrolizumab for treatment of refractory melanoma. Lancet Oncol..

[74] Poole R.M. (2014). Pembrolizumab: First global approval. Drugs.

[75] Minami H., Doi T., Toyoda M., Imamura Y., Kiyota N., Mitsuma A., Shimokata T., Naito Y., Matsubara N., Tajima T. (2020). Phase I study of the anti-PD-1 antibody spartalizumab (PDR001) in Japanese patients with advanced malignancies. Cancer Sci..

[76] Naing A., Gainor J.F., Gelderblom H., Forde P.M., Butler M.O., Lin C.C., Sharma S., Ochoa de Olza M., Varga A., Taylor M. (2020). A first-in-human phase 1 dose escalation study of spartalizumab (PDR001), an anti-PD-1 antibody, in patients with advanced solid tumors. J. Immunother. Cancer.

[77] Migden M.R., Rischin D., Schmults C.D., Guminski A., Hauschild A., Lewis K.D., Chung C.H., Hernandez-Aya L., Lim A.M., Chang A.L.S. (2018). PD-1 Blockade with Cemiplimab in Advanced Cutaneous Squamous-Cell Carcinoma. N. Engl. J. Med..

[78] Sidaway P. (2018). Cemiplimab effective in cutaneous SCC. Nat. Rev. Clin. Oncol..

[79] Kaufman H.L., Russell J., Hamid O., Bhatia S., Terheyden P., D’Angelo S.P., Shih K.C., Lebbe C., Linette G.P., Milella M. (2016). Avelumab in patients with chemotherapy-refractory metastatic Merkel cell carcinoma: A multicentre, single-group, open-label, phase 2 trial. Lancet Oncol..

[80] Sidaway P. (2016). Skin cancer: Avelumab effective against Merkel-cell carcinoma. Nat. Rev. Clin. Oncol..

[81] Rosenberg J.E., Hoffman-Censits J., Powles T., van der Heijden M.S., Balar A.V., Necchi A., Dawson N., O’Donnell P.H., Balmanoukian A., Loriot Y. (2016). Atezolizumab in patients with locally advanced and metastatic urothelial carcinoma who have progressed following treatment with platinum-based chemotherapy: A single-arm, multicentre, phase 2 trial. Lancet.

[82] Sidaway P. (2016). Urological cancer: Atezolizumab effective against advanced disease. Nat. Rev. Clin. Oncol..

[83] Antonia S., Goldberg S.B., Balmanoukian A., Chaft J.E., Sanborn R.E., Gupta A., Narwal R., Steele K., Gu Y., Karakunnel J.J. (2016). Safety and antitumour activity of durvalumab plus tremelimumab in non-small cell lung cancer: A multicentre, phase 1b study. Lancet Oncol..

[84] Brower V. (2016). Anti-PD-L1 inhibitor durvalumab in bladder cancer. Lancet Oncol..

[85] Chauhan K., Jandu J.S., Goyal A., Bansal P., Al-Dhahir M.A. (2020). Rheumatoid Arthritis. StatPearls.

[86] Weyand C.M., Goronzy J.J. (2020). The immunology of rheumatoid arthritis. Nat. Immunol..

[87] Justiz Vaillant A.A., Goyal A., Bansal P., Varacallo M. (2020). Systemic Lupus Erythematosus. StatPearls.

[88] Tsokos G.C. (2020). Autoimmunity and organ damage in systemic lupus erythematosus. Nat. Immunol..

[89] Li R., Patterson K.R., Bar-Or A. (2018). Reassessing B cell contributions in multiple sclerosis. Nat. Immunol..

[90] Tafti D., Ehsan M., Xixis K.L. (2020). Multiple Sclerosis. StatPearls.

[91] Taylor P.C., Feldmann M. (2009). Anti-TNF biologic agents: Still the therapy of choice for rheumatoid arthritis. Nat. Rev. Rheumatol..

[92] Kim G.W., Lee N.R., Pi R.H., Lim Y.S., Lee Y.M., Lee J.M., Jeong H.S., Chung S.H. (2015). IL-6 inhibitors for treatment of rheumatoid arthritis: Past, present, and future. Arch. Pharm. Res..

[93] Kasama T., Isozaki T., Takahashi R., Miwa Y. (2016). Clinical effects of tocilizumab on cytokines and immunological factors in patients with rheumatoid arthritis. Int. Immunopharmacol..

[94] Abbasi M., Mousavi M.J., Jamalzehi S., Alimohammadi R., Bezvan M.H., Mohammadi H., Aslani S. (2019). Strategies toward rheumatoid arthritis therapy; the old and the new. J. Cell Physiol..

[95] Kitching A.R., Anders H.J., Basu N., Brouwer E., Gordon J., Jayne D.R., Kullman J., Lyons P.A., Merkel P.A., Savage C.O.S. (2020). ANCA-associated vasculitis. Nat. Rev. Dis. Primers.

[96] Kronbichler A., Lee K.H., Denicolo S., Choi D., Lee H., Ahn D., Kim K.H., Lee J.H., Kim H., Hwang M. (2020). Immunopathogenesis of ANCA-Associated Vasculitis. Int. J. Mol. Sci..

[97] Swaak A.J., Huysen V., Nossent J.C., Smeenk R.J. (1990). Antinuclear antibody profiles in relation to specific disease manifestations of systemic lupus erythematosus. Clin. Rheumatol..

[98] Cabral A.R., Alarcon-Segovia D. (1997). Autoantibodies in systemic lupus erythematosus. Curr. Opin. Rheumatol..

[99] Cortini A., Bembich S., Marson L., Cocco E., Edomi P. (2019). Identification of novel non-myelin biomarkers in multiple sclerosis using an improved phage-display approach. PLoS ONE.

[100] Vandormael P., Verschueren P., De Winter L., Somers V. (2017). cDNA phage display for the discovery of theranostic autoantibodies in rheumatoid arthritis. Immunol. Res..

[101] Wu F.L., Lai D.Y., Ding H.H., Tang Y.J., Xu Z.W., Ma M.L., Guo S.J., Wang J.F., Shen N., Zhao X.D. (2019). Identification of Serum Biomarkers for Systemic Lupus Erythematosus Using a Library of Phage Displayed Random Peptides and Deep Sequencing. Mol. Cell Proteom..

[102] Russo N., Wang X., Liu M., Banerjee R., Goto M., Scanlon C., Metwally T., Inglehart R.C., Tsodikov A., Duffy S. (2013). A novel approach to biomarker discovery in head and neck cancer using an autoantibody signature. Oncogene.

[103] Dong X., Yang M., Sun H., Lu J., Zheng Z., Li Z., Zhong L. (2013). Combined measurement of CA 15-3 with novel autoantibodies improves diagnostic accuracy for breast cancer. Onco Targets Ther..

[104] Wang X., Yu J., Sreekumar A., Varambally S., Shen R., Giacherio D., Mehra R., Montie J.E., Pienta K.J., Sanda M.G. (2005). Autoantibody signatures in prostate cancer. N. Engl. J. Med..

[105] Larman H.B., Zhao Z., Laserson U., Li M.Z., Ciccia A., Gakidis M.A., Church G.M., Kesari S., Leproust E.M., Solimini N.L. (2011). Autoantigen discovery with a synthetic human peptidome. Nat. Biotechnol..

[106] Lindpaintner K. (2002). The impact of pharmacogenetics and pharmacogenomics on drug discovery. Nat. Rev. Drug Discov..

[107] Dopazo J. (2014). Genomics and transcriptomics in drug discovery. Drug Discov. Today.

[108] Van Rensburg I.C., Loxton A.G. (2015). Transcriptomics: The key to biomarker discovery during tuberculosis?. Biomark. Med..

[109] Petricoin E.F., Zoon K.C., Kohn E.C., Barrett J.C., Liotta L.A. (2002). Clinical proteomics: Translating benchside promise into bedside reality. Nat. Rev. Drug Discov..

[110] Kavallaris M., Marshall G.M. (2005). Proteomics and disease: Opportunities and challenges. Med. J. Aust..

[111] Hanash S. (2003). Disease proteomics. Nature.

[112] Nishimura T., Ogiwara A., Fujii K., Kawakami T., Kawamura T., Anyouji H., Kato H. (2005). Disease proteomics toward bedside reality. J. Gastroenterol..

[113] Yates J.R., Ruse C.I., Nakorchevsky A. (2009). Proteomics by mass spectrometry: Approaches, advances, and applications. Annu. Rev. Biomed. Eng..

[114] Robinson M.R., Miller R.A., Spellman D.S. (2019). Mass Spectrometry-Based Biomarkers in Drug Development. Adv. Exp. Med. Biol..

[115] Kaufmann H., Bailey J.E., Fussenegger M. (2001). Use of antibodies for detection of phosphorylated proteins separated by two-dimensional gel electrophoresis. Proteomics.

[116] Chaga G.S. (2008). Antibody arrays for determination of relative protein abundances. Methods Mol. Biol..

[117] Xu Z.W., Zhang T., Song C.J., Li Q., Zhuang R., Yang K., Yang A.G., Jin B.Q. (2008). Application of sandwich ELISA for detecting tag fusion proteins in high throughput. Appl. Microbiol. Biotechnol..

[118] Rimm D.L., Camp R.L., Charette L.A., Costa J., Olsen D.A., Reiss M. (2001). Tissue microarray: A new technology for amplification of tissue resources. Cancer J..

[119] Au N.H., Gown A.M., Cheang M., Huntsman D., Yorida E., Elliott W.M., Flint J., English J., Gilks C.B., Grimes H.L. (2004). P63 expression in lung carcinoma: A tissue microarray study of 408 cases. Appl Immunohistochem. Mol. Morphol..

[120] Imai S., Nagano K., Yoshida Y., Okamura T., Yamashita T., Abe Y., Yoshikawa T., Yoshioka Y., Kamada H., Mukai Y. (2011). Development of an antibody proteomics system using a phage antibody library for efficient screening of biomarker proteins. Biomaterials.

[121] Imai S., Mukai Y., Nagano K., Shibata H., Sugita T., Abe Y., Nomura T., Tsutsumi Y., Kamada H., Nakagawa S. (2006). Quality enhancement of the non-immune phage scFv library to isolate effective antibodies. Biol. Pharm. Bull..

[122] Nagano K., Imai S., Mukai Y., Nakagawa S., Abe Y., Kamada H., Tsunoda S., Tsutsumi Y. (2009). Rapid isolation of intrabody candidates by using an optimized non-immune phage antibody library. Pharmazie.

[123] Nagano K., Maeda Y., Kanasaki S., Watanabe T., Yamashita T., Inoue M., Higashisaka K., Yoshioka Y., Abe Y., Mukai Y. (2014). Ephrin receptor A10 is a promising drug target potentially useful for breast cancers including triple negative breast cancers. J. Control. Release.

[124] Carey L., Winer E., Viale G., Cameron D., Gianni L. (2010). Triple-negative breast cancer: Disease entity or title of convenience?. Nat. Rev. Clin. Oncol..

[125] Podo F., Buydens L.M., Degani H., Hilhorst R., Klipp E., Gribbestad I.S., Van Huffel S., van Laarhoven H.W., Luts J., Monleon D. (2010). Triple-negative breast cancer: Present challenges and new perspectives. Mol. Oncol..

[126] Pal S.K., Childs B.H., Pegram M. (2011). Triple negative breast cancer: Unmet medical needs. Breast Cancer Res. Treat..

[127] Nagano K., Kanasaki S., Yamashita T., Maeda Y., Inoue M., Higashisaka K., Yoshioka Y., Abe Y., Mukai Y., Kamada H. (2013). Expression of Eph receptor A10 is correlated with lymph node metastasis and stage progression in breast cancer patients. Cancer Med..

[128] Taki S., Kamada H., Inoue M., Nagano K., Mukai Y., Higashisaka K., Yoshioka Y., Tsutsumi Y., Tsunoda S. (2015). A Novel Bispecific Antibody against Human CD3 and Ephrin Receptor A10 for Breast Cancer Therapy. PLoS ONE.

[129] Nagano K., Yamashita T., Inoue M., Higashisaka K., Yoshioka Y., Abe Y., Mukai Y., Kamada H., Tsutsumi Y., Tsunoda S. (2014). Eph receptor A10 has a potential as a target for a prostate cancer therapy. Biochem. Biophys. Res. Commun..

[130] Nagano K., Imai S., Zhao X., Yamashita T., Yoshioka Y., Abe Y., Mukai Y., Kamada H., Nakagawa S., Tsutsumi Y. (2015). Identification and evaluation of metastasis-related proteins, oxysterol binding protein-like 5 and calumenin, in lung tumors. Int. J. Oncol..

[131] Yamashita T., Nagano K., Kanasaki S., Maeda Y., Furuya T., Inoue M., Nabeshi H., Yoshikawa T., Yoshioka Y., Itoh N. (2012). Annexin A4 is a possible biomarker for cisplatin susceptibility of malignant mesothelioma cells. Biochem. Biophys. Res. Commun..

[132] Merchant A.M., Zhu Z., Yuan J.Q., Goddard A., Adams C.W., Presta L.G., Carter P. (1998). An efficient route to human bispecific IgG. Nat. Biotechnol..

[133] Yu J., Wang W., Huang H. (2019). Efficacy and safety of bispecific T-cell engager (BiTE) antibody blinatumomab for the treatment of relapsed/refractory acute lymphoblastic leukemia and non-Hodgkin’s lymphoma: A systemic review and meta-analysis. Hematology.

[134] Hosseini S.S., Khalili S., Baradaran B., Bidar N., Shahbazi M.A., Mosafer J., Hashemzaei M., Mokhtarzadeh A., Hamblin M.R. (2020). Bispecific monoclonal antibodies for targeted immunotherapy of solid tumors: Recent advances and clinical trials. Int. J. Biol. Macromol..

[135] McGuinness B.T., Walter G., FitzGerald K., Schuler P., Mahoney W., Duncan A.R., Hoogenboom H.R. (1996). Phage diabody repertoires for selection of large numbers of bispecific antibody fragments. Nat. Biotechnol..

[136] Fagete S., Botas-Perez L., Rossito-Borlat I., Adea K., Gueneau F., Ravn U., Rousseau F., Kosco-Vilbois M., Fischer N., Hartley O. (2017). Dual display: Phage selection driven by co-engagement of two targets by two different antibody fragments. Protein Eng. Des. Sel..

[137] Luthra A., Langley D.B., Schofield P., Jackson J., Abdelatti M., Rouet R., Nevoltris D., Mazigi O., Crossett B., Christie M. (2019). Human Antibody Bispecifics through Phage Display Selection. Biochemistry.

[138] Fujiwara D., Ye Z., Gouda M., Yokota K., Tsumuraya T., Fujii I. (2010). Selection of inhibitory peptides for Aurora-A kinase from a phage-displayed library of helix-loop-helix peptides. Bioorg. Med. Chem. Lett..

[139] Fujiwara D., Fujii I. (2013). Phage selection of peptide “microantibodies”. Curr. Protoc. Chem. Biol..

[140] Nagano K., Tsutsumi Y. (2016). Development of novel drug delivery systems using phage display technology for clinical application of protein drugs. Proc. Jpn. Acad. Ser. B Phys. Biol. Sci..

